# How well do physical activity questions perform? A European cognitive testing study

**DOI:** 10.1186/s13690-015-0109-5

**Published:** 2015-12-01

**Authors:** J. D. Finger, L. Gisle, H. Mimilidis, C. Santos-Hoevener, E. K. Kruusmaa, A. Matsi, L. Oja, M. Balarajan, M. Gray, A. L. Kratz, C. Lange

**Affiliations:** Robert Koch Institute (RKI), Department of Epidemiology and Health Monitoring, Berlin, Germany; Scientific Institute of Public Health (IPH), Brussels, Belgium; National Institute for Health Development (NIHD), Tallinn, Estonia; National Centre for Social Research (NatCen), London, UK

**Keywords:** International physical activity questionnaire, National health interview survey, Cognitive interviewing, Europe

## Abstract

**Background:**

Only few studies have focused on the cognitive processes of the respondents that are involved when answering physical activity questionnaires (PAQs). This study aimed at examining whether two PAQs work as intended with different segments of the survey population in different cultural settings in Europe.

**Methods:**

The International Physical Activity Questionnaire - Short Form (IPAQ-SF) and the US National Health Interview Survey - Adult Core Physical Activity Questionnaire (NHIS-PAQ) were tested in Belgium, Estonia, Germany and the UK using a standardized cognitive interviewing procedure. IPAQ-SF measures total vigorous physical activity (PA), moderate PA, walking and sitting. NHIS-PAQ measures leisure-time vigorous PA, light and moderate PA and muscle-strengthening PA. In total 62 persons completed cognitive interviews, at least 15 interviews were conducted in each country.

**Results:**

Both PAQs performed as intended with young and high-skilled persons and those having a regular exercise schedule. For the others, however, the testing revealed that problems occurred with both PAQs relating to understanding the concepts of ‘(light and) moderate’ and ‘vigorous’ PA, classifying activities into the provided answer options of different PA intensities, recalling instances of ‘normal’ activities such as walking and sitting, and calculating the total duration of more than one activity or instance of an activity. The revealed problems with the questionnaires were quite similar in different countries; profound cultural differences were not observed.

**Conclusions:**

Both questionnaires were difficult to answer for many respondents and rather user-unfriendly. They are designed to measure an exactness of PA quantity (frequency and duration) and intensity which would be desirable to obtain from a scientific point of view; however, respondents can hardly provide this information for cognitive reasons. Studies investigating the respondents’ perspective are useful for improving physical activity information based on self-reports.

**Electronic supplementary material:**

The online version of this article (doi:10.1186/s13690-015-0109-5) contains supplementary material, which is available to authorized users.

## Background

Physical activity (PA) is one of the major determinants of health [1, 2]. Hence, it is recommended that PA information is collected in the framework of the European Public Health Program aiming at a harmonized health monitoring system, more specifically for the European Core Health Indicators (ECHI) [3]. PA is foreseen to be assessed via self-reports within the second wave of the European Health Interview Survey (EHIS), a common EU instrument developed by Eurostat [4].

Health interview survey (HIS) questionnaires that are used in population health monitoring systems are generally extensive – data on many health topics are obtained – thus, the sub-modules must be concise and easy to answer in different cultural settings. However, designing concise and intelligible questions on PA is a challenge, since it is a multifaceted construct involving different dimensions (frequency, duration and intensity), types (aerobic, muscle-strengthening and muscular-endurance activities) and domains (work, transportation, household and leisure activities) [5, 6]. In past decades, PA research mainly focused on health-enhancing, moderate-to-vigorous leisure-time PA [7]. Recently, studies have been using more holistic or domain-specific questionnaires to measure ‘total’ PA level. Yet, there is no consensus on a reference standard as all established instruments have certain validity limitations [8–10]. Many studies have investigated the construct validity of PA questionnaires (PAQs) by comparing self-reported and objectively measured PA information. Only few studies have focused on the cognitive processes of the respondents that are involved when answering PAQs [11]. Answering quantitative questions requires that respondents understand what the questions refer to and which behavior they are supposed to report. They have to recall relevant instances from memory, to decide whether or not the instances occurred in a given reference period and if the reconstructed instances reflect their usual behavior or not. Finally, respondents have to re-adjust their report into the provided response options and may further revise it for reasons of social desirability [12–14]. Cognitive interviewing is a qualitative method used to evaluate the respondents’ understanding of the questions and answer mechanisms, to identify problems in the design of survey questions and possible reasons for misclassification bias [15].

This article aims at investigating whether two PAQs work as intended with different segments of the survey population in different cultural settings in Europe. More specifically, it aims at identifying major problems with the questionnaires, investigating the respondents’ thought process and developing recommendations to adapt PA questions to the requirements of a multinational HIS study.

## Methods

Four research institutes from Belgium, Estonia, Germany and the UK collaborated in an international cognitive testing study for the improvement of the EHIS wave one (2006–2009) questionnaire modules on physical activity, mental health and alcohol consumption. All four research institutes participated in a coordination meeting in Berlin in January 2011 which served to harmonize the cognitive interviewing methodology. Standardized materials and methods such as translation protocols for translating the source questionnaires, cognitive interview probe sheets, materials for the interviewers training and sheets for the data analysis were developed. The probe sheet and the data analysis template are presented in the Additional file 1 and 2.

### Study sample

The recruited individuals lived in the areas of Berlin, Brussels, London/Lancashire/Nottinghamshire and Tallinn, respectively. At least 15 participants were selected in each country using a common age‐sex roster with the age-group strata 15–19, 20–39, 40–59 and 60+ years. The total study sample comprised 62 participants who completed an interview, 33 men and 29 women. The study was approved by the Board of the Federal Commissioner for Data Protection Berlin, Germany. Respondents were informed about the study objectives, the interview process, and the applicable data protection guidelines (anonymous data processing and record keeping). Each participant gave informed written consent before enrolling for the study. The respondents received an incentive for their study participation.

### Cognitive interviews

Cognitive testing is a systematic method to obtain information on the respondents’ thought process in interpreting a survey question and arriving at an answer. ‘Think-alouds’ and ‘probes’ are habitually used as tools to reveal and document the thought process. The qualitative data are then analysed to identify problems with survey questions [15, 16].

**Fig. 1:**
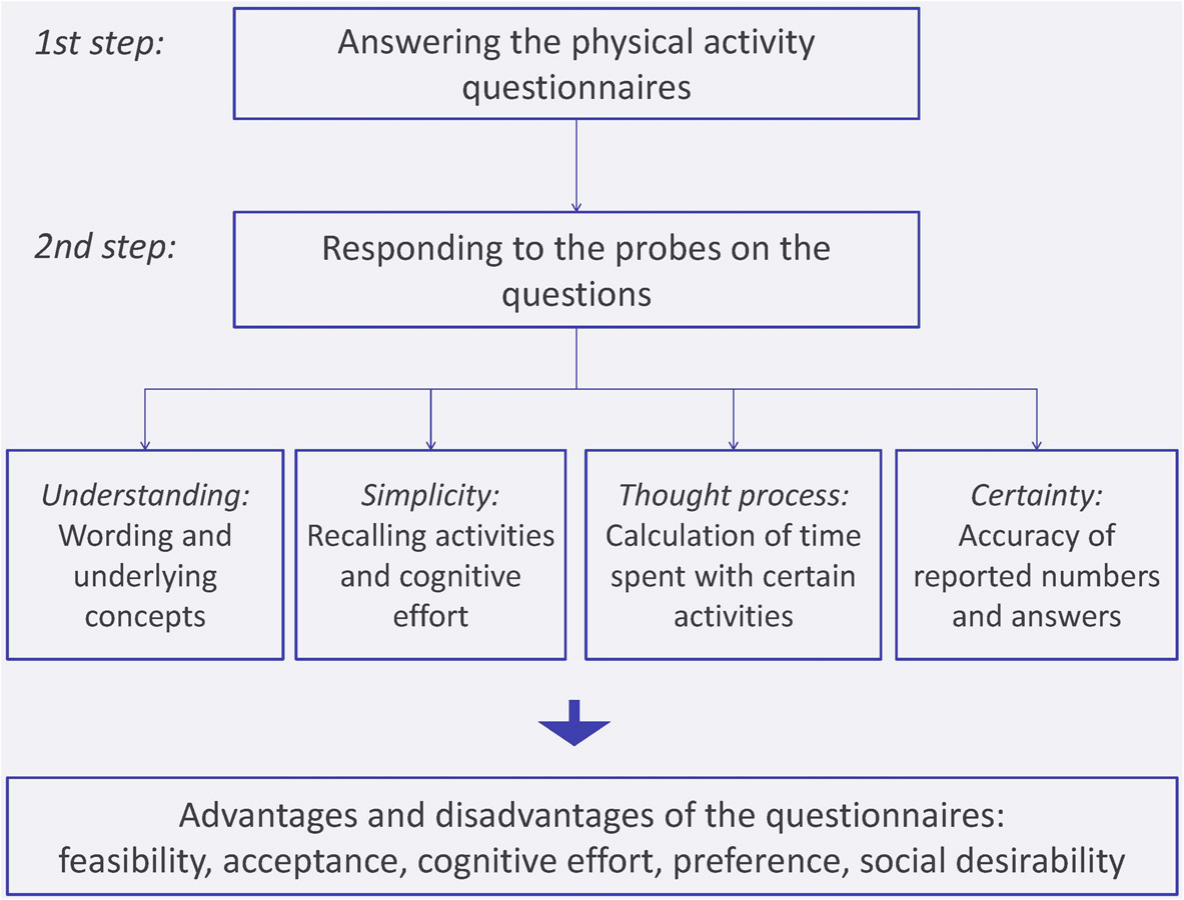
Standardized cognitive testing procedure

The face-to-face interviews were conducted by trained interviewers in the localities of the participating research institutes or at the respondent’s home following a standardized procedure (Fig. 1). The interviews were conducted by three interviewers in the UK, six in Estonia, two in Belgium and three in Germany. In the coordination meeting common interviewer training materials were developed and the interviewer training was carried out in a standardized way. Firstly, the respondents were requested to answer the physical activity questions. Secondly, general and specific questions (probes) were asked in order to collect information on the respondents’ comprehension of the wordings and underlying concepts, the thought process of recalling and classifying activities and calculating frequencies and durations, the simplicity and cognitive effort to answer the questions, the perceived certainty with the reported numbers and answers as well as on issues relating to reference periods, answer options and social desirability. The respondents received a financial incentive after completion of the interview in Belgium, Germany and the UK.

### Source questionnaires

The two questionnaires that were tested were the International Physical Activity Questionnaire - Short Form (IPAQ-SF) and the US National Health Interview Survey - Adult Core Physical Activity Questionnaire (NHIS-PAQ). The NHIS-PAQ has been used since 1998 in the yearly NHIS by the Centers for Disease Control and Prevention (CDC) and may be viewed at http://www.cdc.gov/nchs/nhis/quest_data_related_1997_forward.htm. The IPAQ-SF has been used as a modified version in the first EHIS wave and in the Eurobarometer surveys [17] and may be viewed at http://www.ipaq.ki.se/. The concepts of the questionnaires are described in Table 1 and the full questionnaires can be found in the Additional file 1. IPAQ-SF is a generic instrument and measures total PA in the last 7 days, NHIS-PAQ is a domain-specific instrument which measures leisure-time PA in a usual week. Both PAQs ask for the frequency of days an activity is performed and then for the duration on a ‘typical day’ (NHIS-PAQ) or on ‘one of those days’ (IPAQ-SF) respectively. Those instruments were selected because they represents two different types of questionnaires – IPAQ-SF is a generic instrument and NHIS-PAQ a domain-specific instrument, they are both short-form questionnaires and had been both used in large-scale, population health surveys before and their validity and reliability were evaluated.

**Table 1:** Description of the source questionnaires

Variable code	Description	Answer options
*IPAQ-SF*		
Q1	Frequency of days doing vigorous physical activities for at least 10 min at a time that make you breath much harder in the last 7 days	Days/week
Q2	Usual time spend doing vigorous physical activities on one of those days	Hours/day;
		minutes/day
Q3	Frequency of days doing moderate physical activities for at least 10 min at a time that make you breath somewhat harder in the last 7 days	Days/week
Q4	Usual time spend doing moderate physical activities on one of those days	Hours/day;
		minutes/day
Q5	Frequency of days walking for at least 10 min at a time in the last 7 days	Days/week
Q6	Usual time spend walking on one of those days	Hours/day;
		minutes/day
Q7	Usual time spend sitting on a week day in the last 7 days	Hours/day;
		minutes/day
*NHIS-PAQ*		
Q8	Frequency of doing vigorous leisure-time physical activities for at least 10 min that cause heavy sweating or large increase in breathing or heart rate	Times per day/week/ month/year
Q9	Time spend doing these vigorous leisure-time physical activities each time	Minutes per time
Q10	Frequency of doing light or moderate leisure-time physical activities for at least 10 min that cause only light sweating or a slight to moderate increase in breathing or heart rate	Times per day/week/ month/year
Q11	Time spend doing these light or moderate leisure-time physical activities each time	Minutes per time
Q12	Frequency of doing leisure-time physical activities specifically designed to strengthen your muscles	Times per day/week/ month/year

The translations of instruments were conducted by two independent translators in each country using conceptual translation cards if no validated versions for the target languages were available. The two translators discussed differences between their versions and agreed on a consensus version. The consensus version was checked by a reviewer using a review questionnaire. The translators and the reviewer agreed on a final consensus version in an adjudication panel.

### Data analysis

All interviews were tape-recorded and transcribed. The transcripts were translated into English and entered in the standardized data analysis sheet. The sheet was structured according to the general and specific probes developed for the two PA questionnaires and provided also space to enter the interviewers’ observations. Furthermore, the interviewers’ experiences were assessed in group discussions taking place after the end of field work. The information was documented and analysed. The amount of data was reduced by documenting frequently observed patterns and problems regarding the themes listed above at the end of the section ‘cognitive interviews’. The study coordinators checked the information entered into the data analysis sheets randomly. It was also analysed whether certain patterns and problems were related to specific segments of the population or characteristics of the respondents.

## Results

The characteristics of the respondents are presented in the Table 2. The questionnaires worked as intended with some segments of the survey population, in particular with employed and younger respondents having a regular exercise or working schedule. The questionnaires were more problematic for other segments of the population, in particular for respondents aged 60 years and above and those who did not work regularly (retirees, self-employed or unemployed) or those who performed PA irregularly.

**Table 2:** Characteristics of the respondents

	Belgium	Estonia	Germany	UK	Total
Gender	*n* (%)	*n* (%)	*n* (%)	*n* (%)	*n* (%)
Men	8 (50)	7 (47)	8 (50)	7 (47)	30 (48)
Women	8 (50)	8 (53)	8 (50)	8 (53)	32 (52)
Age group (years)					
15–19	3 (19)	2 (13)	2 (13)	0 (0)	7 (11)
20–39	4 (25)	4 (27)	4 (25)	4 (27)	16 (26)
40–59	4 (25)	4 (27)	4 (25)	3 (20)	15 (24)
60+	5 (31)	5 (33)	6 (38)	8 (53)	24 (39)
Total	16 (100)	15 (100)	16 (100)	15 (100)	62 (100)

Overall 26 respondents indicated that that the NHIS-PAQ was easier to answer, 19 opted for the IPAQ-SF and 20 indicated that both were the same. 15 respondents preferred the NHIS-PAQ, 24 preferred the IPAQ-SF and 20 had no preference. Frequently reported reasons why respondents indicated that the NHIS-PAQ is easier to answer were that they don’t do any LTPA or that it measures exactly what they do, which is LTPA. The NHIS-PAQ was preferred and perceived as being easier to answer by respondents doing sports and exercise in their leisure time, because it measures quite closely what they actually do. People being physically active at work and retirees preferred the IPAQ-SF - although they had to do more calculations - because it better reflects their actual activities (work-related PA, household and gardening), while the NHIS-PAQ made them look physically inactive.

The analysis revealed some major problems with the questionnaires relating to a) the comprehension of the wordings and underlying concepts, b) recalling instances and activities, c) classifying activities into the answer options, d) calculating frequencies and durations and e) sensitivity and social desirability. Case examples for the major problems are illustrated in Table 3.

**Table 3:** Case examples for the major problems

Problem	Case examples
Comprehension of the wording and underlying concepts	- R (female 81, DE), NHIS-PAQ: “As I am a retired person, I have actually always leisure time, I can do everything how I want it to, except my household works or appointments, as today…”.
	- R (male 45, DE), NHIS-PAQ: There is no definition of ‘leisure time’ for him because of his job, which is not a usual one; he is not a typical employee (self-employed). Problematic, because for him there is not a difference between work and leisure time.
	- R (male 74, DE), NHIS-PAQ: “As a retired person you arrange your duties that they are distributed along the whole day, so I virtually do not have any leisure time [laughed].”
	- R (male 70, DE), NHIS-PAQ: “You have to distinguish between an employed and a retired person. For me (retired) everything is leisure time. When I used to work, it was the time after work”.
	- R (female 41, DE), IPAQ-SF: R had problems to understand the question, asked for response categories. She said the question was okay to answer, but while answering the questionnaire she really had problems to understand it.
Recalling instances and activities	- R (male 31, UK): R says that IPAQ-SF is harder to answer than NHIS-PAQ: It is a lot more to remember, it is wordy and difficult to follow what is being included and excluded, and it is difficult to make a quick calculation to work out the activity duration.
	- R (male 74, DE): NHIS-PAQ was easy to answer for him because he does not do these kind of activities.
	- R (male 21, DE), NHIS-PAQ, interviewer observation: R has to think for a long time and wonders whether he should include work (until I tell him, that this part of the questionnaire is just about leisure time).
	- R (male 17, DE), IPAQ-SF: R seems a little worried about the difference between ‘last seven days’ and ‘usually’; he was on holidays for skiing and is therefore quite confused about what to answer.
	- R (female 59, DE), IPAQ-SF: She only thought about work-related activities, so the duration of vigorous PA might be too short; IPAQ-SF moderate activities: Her answer deals with cycling in leisure time, during the interview she said, she is going to work by bike every day; so here her answer might be an underestimation.
Classifying activities into the answer options	- R (female 26, UK): R felt that she contradicted herself when talking about ‘vigorous’ in NHIS-PAQ to which she gave different answers in IPAQ-SF. The way the descriptions were presented, made her think of the same term in 2 different ways. She does not get into a heavy sweat [NHIS-PAQ] but does do activities that need ‘hard physical effort’ as described in IPAQ-SF. R did not understand ‘moderate,’ as in both Sets, they also had different definitions. For ‘moderate activities’ she answered ‘yes’ in NHIS-PAQ and ‘no’ in IPAQ-SF.
	- R (female 26, UK), IPAQ-SF: “If you’re trying to identify the types of exercise I do, there surely is a simpler way of finding-out what and how regularly I exercise (…). It seemed like I had to slot them into the descriptions, which wasn’t easy to do. I wasn’t sure I was answering them correctly, even thought I was sure of the activity I was doing, I didn’t know if I was answering them appropriately or accurately”.
	- R (female 33, EE), IPAQ-SF: R is confused about what to consider and what not: “In some questions it is necessary to take walking into account and in others not. Is sportive walking included to walking or not? All depends on the intensity, after all”.
	- R (female 42, BE), IPAQ-SF: “The distinctions that have to be made are difficult because everything is linked in one activity”.
	- R (female 62, UK), IPAQ-SF: R included a vigorous walk in Q1. This made it difficult for her to answer Q3; without the walks she had to say that she did no moderate activity. When she came to Q5 she thought she answered wrong and was confused; her vigorous walk potentially could fit in either section.
	- R (female 73, DE), NHIS-PAQ: R talks about her gymnastics, thought about sweating, then answers Q8. R describes again her gymnastics and answers Q10. R describes again her gymnastics that are also strengthening, she seems annoyed that questions are very similar, and answers Q12.
	- R (female 26, UK): She was including all of her physical activities when she was answering NHIS-PAQ; she was not restricting herself to leisure activities. She was also including work.
Calculating frequencies and durations	- R (male 43, UK), NHIS-PAQ, interviewer observation: The problem that I had was the R didn’t answer the questions in the way they were intended. The first difficulty was fitting in what the R was telling me to the answer options available. The more he explains the more difficult it is to answer the questions. He goes to the gym 3 times a week for an hour at a time and goes jogging every day between 30 and 45 min. He is not sure to say 1h 45mins a day or 45min a day (to use a day at the gym or a day when he doesn’t go to the gym.). The second difficulty was the R answering that he did light to moderate exercises all day everyday, he failed to give me time periods.
	*-* R (female 42, BE), IPAQ-SF: “I can’t remember what I answered and I can’t even tell how I calculated”.
	- R (female 60, BE), IPAQ-SF: “Everything is relative what we can call vigorous. For me vacuuming is vigorous, I’ve done it 4 times this week, for others it is of course moderate”.
	- R (female 81, DE), IPAQ-SF: “I just approximated the time for sitting and walking, it is so difficult to estimate because it is s.th. so normal”.

### Comprehension of the wordings and underlying concepts

Many respondents had difficulties to understand the terms of ‘(light and) moderate’ and ‘vigorous’ PA in both PAQs. Some respondents had never heard these words before and did not know what they mean. Other respondents misunderstood the meaning of some terms. They thought about activities which are stressful and mentally demanding but do not involve extra physical effort. Respondents who correctly understood the questions often did not find it intuitive to think in those PA intensity categories about the activities they do, for instance because one specific activity involved episodes of different intensity levels. The examples given for the PA intensity levels in the IPAQ-SF were generally perceived as useful. In the NHIS-PAQ respondents had even greater problems to understand which activities they should think of. Some respondents mentioned that the question wording, in particular that of the IPAQ-SF, was too long and confusing. Some respondents had to read the explanative text preceding the question twice to grasp the information. Other respondents skipped reading parts of the text which resulted in misunderstandings. Some respondents had problems to define their leisure time for answering the NHIS-PAQ; this was particularly difficult for unemployed, retired or self-employed persons.

### Recalling instances and activities

It appeared that activities on which respondents ‘usually do not take track of’ such as sitting (IPAQ-SF), walking (IPAQ-SF) and to some extent (light to) moderate PA (IPAQ-SF and NHIS-PAQ) were reported to be in general more difficult to recall than vigorous PA. Respondents mainly classified sports and exercise as vigorous PA which is usually ‘planned’, can be ‘controlled’ and follow a ‘regular schedule’, what makes it easier to recall. In contrast, sitting and walking were perceived as ‘normal’ activities that are ‘fragmented’, ‘confuse’ and performed without ‘conscious recognition’, what make them more difficult to remember. As a result, many respondents had problems to recall the instances of those activities and were not able to provide an exact overall duration. To ask for exact hours and minutes for activities respondents only can provide rough estimates, led in some cases to the unwillingness to give an answer and to item nonresponse. Some respondents indicated that the *last seven days* reference period (IPAQ-SF) was concrete and helped them to decide which activities they should include. Other respondents mentioned that their activity behaviour in the last seven days was not representative for their usual behaviour for various reasons (seasonality, weather, illness, vacation etc.). Some of those respondents dealt with this problem by ignoring the reference period and reporting their usual behaviour. Others of those respondents reported their activities of the last seven days despite the discrepancy to their usual behaviour. On the other hand, the *usual* behaviour assessed in the NHIS-PAQ was for some respondents difficult to define, in particular if their PA behaviour was irregular (e.g. for retirees).

### Classifying activities into the answer options

Many respondents had problems to decide in which PA section they should include their activities, mainly because they were unable to distinguish between the PA intensity levels in both PAQs. They had difficulties to classify PAs with different intensity levels in one activity. Even if an activity had a more or less homogenous PA intensity level, respondents had difficulties to decide which intensity category fits best and they made arbitrary choices. What was seen as ‘vigorous’ PA among some respondents was sometimes seen as ‘(light and) moderate’ PA among others and vice versa. As a result, many respondents were not sure about the accuracy of their answers, even though they were sure about the activities they were doing. Some respondents included certain activities two or three times. They didn’t grasp the concepts of the PAQs that the questions follow a sequence of sections with decreasing PA intensity and that a specific activity should be reported only once. Other respondents forgot activities or wrongly thought that they should not include them, for instance because they ‘do not sweat’ when they do the activity although it involves physical effort (NHIS-PAQ), or, they only included activities that were explicitly mentioned as examples in the text and forgot similar activities because they were not mentioned (IPAQ-SF).

### Calculating frequencies and durations

IPAQ-SF required more cognitive effort and calculations than the NHIS-PAQ. For some respondents it was difficult to add up the total time spent on different activities or different instances of the same activity. In particular, the total time spent walking and sitting was hard to calculate for many respondents. The NHIS-PAQ required less calculation and was quicker to answer. In the NHIS-PAQ respondents can freely choose in which frequency and duration units they report their light and moderate and vigorous PAs. The interviewers then correctly codify the reports into respective answer options. The interviewers’ feedback was that it was problematic to correctly codify the answer if the respondent reported more than one PA or instance of PA for a certain PA level section. In the IPAQ-SF the respondents had to do the coding themselves. Furthermore, they did not only have to consider all PAs they do in their leisure time like in the NHIS-PAQ, they also had to add all work-related, household and transportation PAs stratified by the different PA dimensions and intensity levels. Doing this in an accurate way was difficult for many respondents and led to a feeling of high uncertainty with the indicated numbers. Some respondents could not remember what they answered, nor tell how they calculated. For this reason, some respondents preferred the NHIS-PAQ because it required less calculation.

### Sensitivity and social desirability bias

The strategy to firstly ask for the frequency of days an activity is performed and then for the duration on a ‘typical day’ (NHIS-PAQ) or on ‘one of those days’ (IPAQ-SF) respectively, may lead to over- or under-reporting. Respondents may choose the day on which they were most or last active as the reference day, instead of referring to an average day. Testing revealed that respondents often chose the day on which they were most active as the reference day. Although many respondents mentioned that social desirability had not influenced their own answers, they generally suspected that ‘other’ people may give an overestimation of their PA behavior. Some respondents reported that they disliked the NHIS-PAQ because it made them look inactive. Others included their work activity when answering the NHIS-PAQ even though they knew that they should not include it. Many respondents preferred the IPAQ-SF because they could report a lot of daily activities, making them look active. Respondents often overestimated the PA intensity level of their activities. For instance they counted daily activities as vigorous PA, although they admitted afterwards that moderate PA would have been more appropriate.

### Country and age differences

The major problems and patterns described above were observed in more than one country. The problem with the distinction of the PA intensity levels was observed in all countries. In Belgium and Germany it was difficult to find appropriate translations into German and French for the terms of ‘moderate’ and’vigorous’ PA intensity. The muscle-strengthening question of the NHIS-PAQ (Q12) worked widely as intended in all countries; however, in England the example of ‘calisthenics’ was not understood by many respondents. Substantial country-specific problems or cultural differences in administering the PAQs in the different settings in Europe were not observed. In the age group 15 to 19 years of age the questionnaires performed as good as in the other age groups, however, in the age group 60 years and older retired persons reported difficulties to define their leisure time when answering the NHIS-PAQ (see Table 3).

## Discussion

In this comparative cognitive testing study based on samples from diverse cultural settings in four different parts of Europe, it is observed that the two physical activity questionnaires tested worked as intended with some segments of the survey population, in particular with young persons and those having a regularly exercise and working schedule. However, many respondents reported having difficulties filling in both questionnaires. The major problems encountered related to understanding the concepts of ‘(light and) moderate’ and ‘vigorous’ PA, classifying activities into the provided answer options of different PA intensity, recalling instances of ‘normal’ activities such as walking and sitting, and calculating the total duration of more than one activity or instance of an activity. These problems were observed in different countries, age groups and segments of the survey population. However, some participants had more problems than others and some problems were related to specific characteristics of the respondents. For example, respondents aged 60 years and older had more difficulties in the comprehension and answering of the questions than those younger than 60. It could be that activity limitations (hearing and seeing conditions) and cognitive impairment, which increase with increasing age [18], play a role, but also the regularity of physical activity behavior – retired respondents reported more often than working-age respondents that their activities are irregularly and thus difficult to recall. Hence, the information bias is unlikely to be randomly distributed, and it must be assumed that differential misclassification bias occur when administering the PAQs. The greatest misclassification bias is likely to occur in the sections of (light and) moderate PA, walking and sitting. Studies which have focused on the validation of PA questionnaires have quite consistently shown that the correlation coefficients of the construct validity comparing PA questions against accelerometers or pedometers were higher for vigorous PA than for moderate PA, and walking and sitting showed the lowest coefficients [8, 9, 19]. In line with these findings, we observed that the uncertainty in selecting the intended activities was highest in the (light and) moderate PA sections and that the uncertainty in recalling instances and calculating the total duration was highest for sitting and walking. Berrigan *et al*. carried out a cognitive testing study on the NHIS-PAQ and reported in line with our observations that respondents had problems with the vague terms of ‘vigorous’ and ‘light and moderate’ PA, with recalling information and with estimating frequency and duration across the set of items of activities [20]. Furthermore, Altschuler et al. in their cognitive testing study also reported that respondents had problems with the PA intensity definitions and that respondents understood ‘intensity’ in terms of emotional or psychological intensity [11]. Among women and older people household activities are often included in the moderate PA section. Hence, PA recommendation compliance of women and older people to a significant extent is attributable to household activities. It was questioned however, whether the intensity of household activities is sufficient to bring about all of the health benefits normally associated with meeting the PA recommendations [21]. The problem of distinguishing ‘(light and) moderate’ and ‘vigorous’ PAs has been subject of discussions in the process of designing PAQs and developing PA guidelines for a long time. For instance the CDC published new descriptions for moderate and vigorous PA in the *2008 Physical Activity Guidelines for Americans* [22]. However, this problem has never been fully solved. Based on the findings of this study and considering the evidence from other studies, it must generally be questioned if the distinction between different PA intensity levels in PA self-reports makes sense. Respondents have to make difficult transformations to divide their PAs into intensity categories, and many respondents were apparently not able to do this in an accurate way and reported activities more than once or forgot about other activities.

The finding that respondents had severe problems to accurately quantify the total duration for certain sets of PAs complies with the findings of a recent review on studies that focus on the validity of PAQs [23]. The authors discuss that the absolute validity of PAQs to quantify individuals’ PA habits is in general poor and that it thus would not be recommended to use the continuous PA quantity data for analysis. The only acceptable validity has been presented in form of non-parametric estimators (Pearson and Spearman coefficients) which are based on ranked outcomes [23]. It needs to be discussed whether it is reasonable to obtain detailed continuous PA quantity information with open questions – what puts an extra cognitive burden on the respondents – if the scale level afterwards is reduced for data analyses. Asking for categorical PA quantity information in the first place might be a more efficient and user-friendly alternative. This would particularly make sense for activities people usually do not take track of, such as sitting and walking.

PAQs that are used in general population health surveys also need to consider the cognitive abilities of the people with lower reading and numeracy levels [24]. Furthermore, the questions should avoid wording that combines multiple activities and/or behaviors into a single question that asks respondents about average time spent in multiple activities or across many days [24]. Both, the IPAQ-SF and the NHIS-PAQ infringe upon this principle. Both PAQs are designed to measure an exactness of PA quantity (frequency and duration) and intensity which would be desirable to obtain from a scientific point of view, as these are in theory the most important PA dimensions. Respondents can hardly provide this information for cognitive reasons however. The close adherence to the theoretical PA concepts in the design of the NHIS-PAQ and IPAQ-SF and the lack of consideration of the respondents’ perspective and cognitive abilities make both PAQs rather user-unfriendly and unsuitable for using them in a multinational HIS study.

**Fig. 2:**
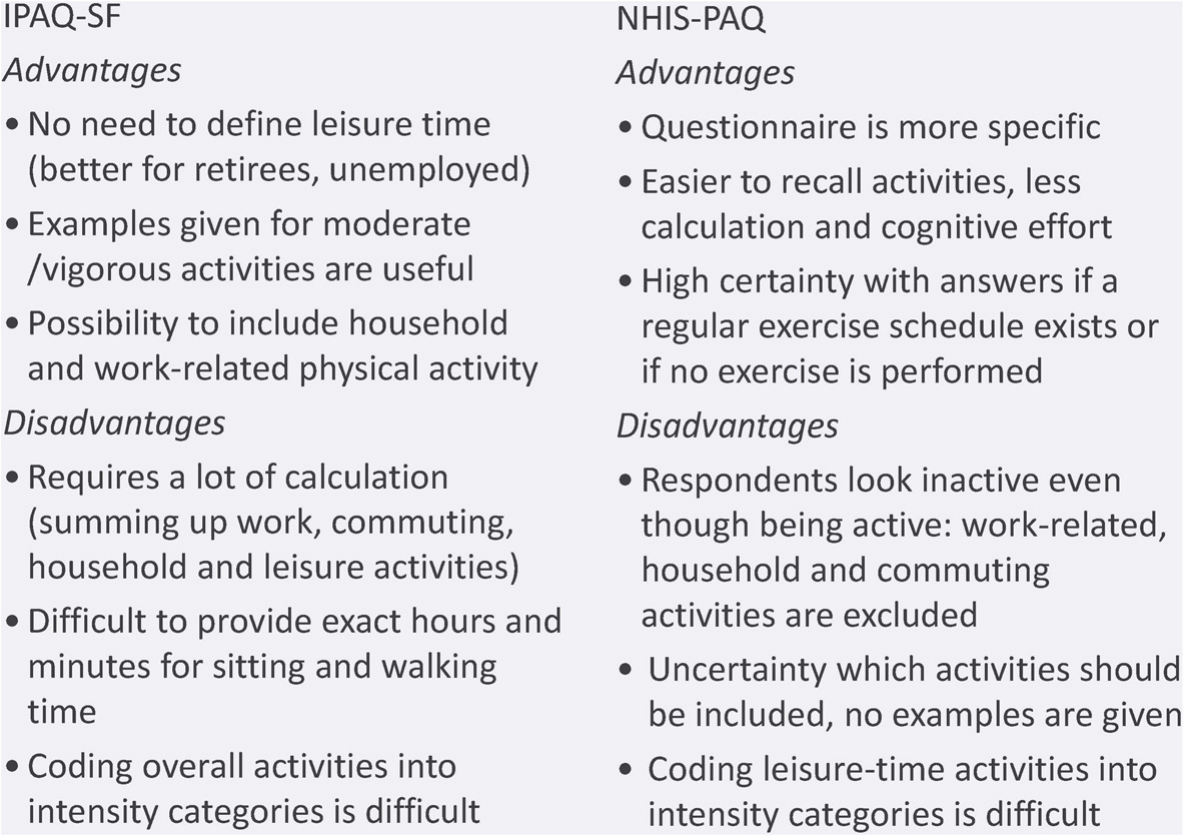
Advantages and disadvantages of the physical activity questionnaires

Figure 2 illustrates the main advantages and disadvantages of the two PAQs. Our recommendation to facilitate answering the PAQs, and hence to avoid item nonresponse and improve reliability, would be: to remove the PA intensity distinction, to assess PAs of different domains separately and to provide ordinal answer categories for indicating PA quantities of activities people usually do not take track of. These adaptations may enhance the simplicity of the PAQs and reduce the cognitive effort for the respondents to answer the PAQs, without losing relevant information. Also, it is important that the PAQ includes a work-related PA section. Otherwise the PA habits of individuals physically active at work but not active in their leisure time would not be adequately represented. Work-related PA is more often reported by persons with low socioeconomic position (SEP) [25]. Thus, PAQs which only consider leisure-time PA (e.g. NHIS-PAQ) will systematically underestimate the PA level of persons with low SEP and may contribute to a limited acceptance with the PAQ in this group.

### Limitations

Cognitive testing methods can present validity and reliability flaws. Validity is challenged when certain problematic features of the questionnaire for the survey population remain undetected in the cognitive testing study or when detected problematic features in the sample interviewed are overestimated in their relevance for the survey population [26]. In addition, the reliability problem that can occur in multicentre cognitive testing studies is that independent groups of researchers may come up with inconsistent findings [26]. We cannot fully exclude the possibility that these problems occurred in this study. However, this study was conducted from different research groups in four countries and the results presented in this article are a summary of the results of four national studies.

As the process of collecting and analysing the data is time consuming in qualitative studies, the sample used in this study is comparatively small compared to those used in quantitative studies. The prevalence of certain patterns in this small sample cannot be generalized to the general population. However, patterns that are observed in cognitive testing studies with small samples usually give a good indication what problems can occur in the general population [20].

## Conclusions

Based on the finding of this study we suggest introducing the following adaptations to improve the PAQs and make them more suitable for respondents in a HIS context: Remove the distinction between intensity levels of activity and define a minimum intensity level of at least moderately vigorous intensity, assess activities of different PA domains separately and cover work-related PA and leisure-time PA (optional: transport-related and household PA), provide for each domain specific examples of activities which should be included, provide categorical answer categories for PA sections that focus on activities people usually don’t take track of such as walking and sitting.

## Additional files

Additional file 1:
**Contains the probe sheet.** (PDF 222 kb) Additional file 2:
**Contains the data analysis sheet.** (XLS 121 kb)
