# Supplementary material for: How well do physical activity questions perform? A European cognitive testing study
Source: Arch Public Health. 2015 Dec 1;73:57. doi: 10.1186/s13690-015-0109-5 (PMC4665945; doi:10.1186/s13690-015-0109-5)
Supplement: Additional file 1: — Contains the probe sheet. (PDF 222 kb) [file 13690_2015_109_MOESM1_ESM.pdf]

QUESTIONS ON PHYSICAL ACTIVITY

PLEASE ASK ALL QUESTIONS OF THIS SECTION FIRST AND THEN PROCEED WITH PROBING

{READ TO ALL}

In the following there are two sections on physical activity, one on leisure-time physical activity and one on total physical activity. One of the aims of this interview is to find out which section works better. Therefore, some of the questions might seem repetitive.

We are now starting with section A about your physical activities in your leisure time and I will let you know when we move to section B.

{READ OUT TO ALL}

The next questions are about physical activities (exercise, sports, physically active hobbies...) that you may do in your LEISURE time.

{ASK ALL}

Q3: How often do you do VIGOROUS leisure-time physical activities for AT LEAST 10 MINUTES that cause HEAVY sweating or LARGE increases in breathing or heart rate?

INTERVIEWER: Responses can be offered in terms of any time unit the respondent volunteers (times per day, per week, per month, or per year)

{READ IF NECESSARY}

How many times per day, per week, per month, or per year do you do these activities?

Never	☐
**1-995 time(s)**	______
Unable to do this type activity	☐
Refused	☐
Don't know	☐

INTERVIEWER: Enter time period for vigorous leisure-time physical activities.

Never	☐	Go to Q4
Per day	☐	ASK Q3a
Per week	☐	ASK Q3a
Per month	☐	ASK Q3a
Per year	☐	ASK Q3a
Unable to do this activity	☐	Go to Q4
Refused	☐	Go to Q4
Don't know	☐	Go to Q4

{ASK ALL}
Q3a: About how long do you do these vigorous leisure-time physical activities each time?

INTERVIEWER: Enter number for length of vigorous leisure-time physical activities.

1-995	______	
Refused	☐	Go to Q4
Don't know	☐	Go to Q4

INTERVIEWER: Enter time period for length of vigorous leisure-time physical activities.
RECORD RESPONDENT'S ANSWER IN EITHER HOURS OR MINUTES

Minutes	☐	Go to Q4
Hours	☐	Go to Q4
Refused	☐	Go to Q4
Don't know	☐	Go to Q4

{ASK ALL}
Q4: How often do you do LIGHT OR MODERATE LEISURE-TIME physical activities for AT LEAST 10 MINUTES that cause ONLY LIGHT sweating or a SLIGHT to MODERATE increase in breathing or heart rate?

{READ IF NECESSARY}
How many times per day, per week, per month, or per year do you do these activities?

Never	☐
**1-995 time(s)**	______
Unable to do this type activity	☐
Refused	☐
Don't know	☐

INTERVIEWER: Enter time period for light or moderate leisure-time physical activities.

Never	☐	Go to Q5
Per day	☐	ASK Q4a
Per week	☐	ASK Q4a
Per month	☐	ASK Q4a
Per year	☐	ASK Q4a
Unable to do this activity	☐	Go to Q5
Refused	☐	Go to Q5
Don't know	☐	Go to Q5

{ASK ALL}
Q4a: About how long do you do these light or moderate leisure-time physical activities each time?

INTERVIEWER: Enter number for length of light or moderate leisure-time physical activities.

1-995	______	
Refused	☐	Go to Q5
Don't know	☐	Go to Q5

INTERVIEWER: Enter time period for length of light or moderate leisure-time physical activities.
RECORD RESPONDENT'S ANSWER IN EITHER HOURS OR MINUTES

Minutes	☐	Go to Q5
Hours	☐	Go to Q5
Refused	☐	Go to Q5
Don't know	☐	Go to Q5

{ASK ALL}
Q5: How often do you do LEISURE-TIME physical activities specifically designed to STRENGTHEN your muscles such as lifting weights or doing calisthenics?
(Include all such activities even if you have mentioned them before.)

{READ IF NECESSARY}
How many times per day, per week, per month, or per year do you do these activities?

Never	☐
**1-995 time(s)**	______
Unable to do this type activity	☐
Refused	☐
Don't know	☐

INTERVIEWER: Enter time period for strengthening activities.

Never	☐	Go to Q6
Per day	☐	Go to Q6
Per week	☐	Go to Q6
Per month	☐	Go to Q6
Per year	☐	Go to Q6
Unable to do this activity	☐	Go to Q6
Refused	☐	Go to Q6
Don't know	☐	Go to Q6

{READ TO ALL}
We have now finished the section A on leisure time physical activity and will move to section B on total physical activity.

{READ OUT TO ALL}
I am going to ask you about the time you spent being physically active in the last 7 days. Please answer each question even if you do not consider yourself to be an active person. Think about the activities you do at work, as part of your house and yard work, to get from place to place, and in your spare time for recreation, exercise or sport.

Q6: Now, think about all the *vigorous* activities which take *hard physical effort* that you did in the last 7 days. Vigorous activities make you breathe much harder than normal and may include heavy lifting, digging, aerobics, or fast bicycling. Think only about those physical activities that you did for at least 10 minutes at a time.

During the **last 7 days**, on how many days did you do **vigorous** physical activities?

Days per week	___	Go to Q7 if zero OTHERWISE ASK Q6a
Don't Know/Not Sure	☐	Go to Q7
Refused	☐	Go to Q7

[**Interviewer clarification**: Think only about those physical activities that you do for at least 10 minutes at a time.]

Q6a: How much time did you usually spend doing **vigorous** physical activities on one of those days?

Hours per day	___ ___	Go to Q7
Minutes per day	___ ___ ___	Go to Q7
Don't Know/Not Sure	☐	Go to Q7
Refused	☐	Go to Q7

[**Interviewer clarification**: Think only about those physical activities you do for at least 10 minutes at a time.]

{READ OUT TO ALL}
Q7: Now think about activities which take *moderate physical effort* that you did in the last 7 days. Moderate physical activities make you breathe somewhat harder than normal and may include carrying light loads, bicycling at a regular pace, or doubles tennis. Do not include walking. Again, think about only those physical activities that you did for at least 10 minutes at a time.

During the **last 7 days**, on how many days did you do **moderate** physical activities?

Days per week	___	Go to Q8 if zero OTHERWISE ASK Q7a
Don't Know/Not Sure	☐	Go to Q8
Refused	☐	Go to Q8

[**Interviewer clarification**: Think only about those physical activities that you do for at least 10 minutes at a time]

Q7a: How much time did you usually spend doing **moderate** physical activities on one of those days?

Hours per day	___ ___	Go to Q8
Minutes per day	___ ___ ___	Go to Q8
Don't Know/Not Sure	☐	Go to Q8
Refused	☐	Go to Q8

[**Interviewer clarification**: Think only about those physical activities that you do for at least 10 minutes at a time.]

{READ OUT TO ALL}
Q8: Now think about the time you spent walking in the last 7 days. This includes at work and at home, walking to travel from place to place, and any other walking that you might do solely for recreation, sport, exercise, or leisure.

During the **last 7 days**, on how many days did you **walk** for at least 10 minutes at a time?

Days per week	___	Go to Q9 if zero OTHERWISE ASK Q8a
Don't Know/Not Sure	☐	Go to Q9
Refused	☐	Go to Q9

[**Interviewer clarification**: Think only about the walking that you do for at least 10 minutes at a time.]

Q8a: How much time did you usually spend **walking** on one of those days?

Hours per day	___ ___	Go to Q9
Minutes per day	___ ___ ___	Go to Q9
Don't Know/Not Sure	☐	Go to Q9
Refused	☐	Go to Q9

{READ OUT TO ALL}
Now think about the time you spent sitting on week days during the last 7 days. Include time spent at work, at home, while doing course work, and during leisure time. This may include time spent sitting at a desk, visiting friends, reading or sitting or lying down to watch television.

Q9: During the last 7 days, how much time did you usually spend ***sitting*** on a **week day**?

Hours per day	___ ___	Go to next part
Minutes per day	___ ___ ___	Go to next part
Don't Know/Not Sure	☐	Go to next part
Refused	☐	Go to next part

[**Interviewer clarification**: Include time spent lying down (awake) as well as sitting]

Physical Activity Section
Aims:

- What set of questions was easier to answer?
- Do respondents understand the underlying concepts of the questions?
- How do Rs remember their physical activity level?
- Do Rs understand the wording of respective questions?
- Was there any question that made Rs feel uncomfortable?
- How do Rs decide on their answer? (I.e. is it compared to others, compared to their needs/wants, etc?)
- Are Rs answering in a truthful way?

1. General probes

- If you think of the two different sections (leisure-time versus total activity), which set of questions was easier for you to answer and why? Examples
- Which set of questions did you prefer answering? Read out…
    1. Set A: Leisure-time physical activity
    2. Set B: Total physical activity
    3. Or do you not have a preference?

INTERVIEWER: EXPLORE THE REASONS FOR THE PREFERRED SET OF QUESTIONS.

- In general was it easy or hard to remember your physical activities?

2. General probes for the leisure-time physical activity questions (section A)

- How were these questions for you?
- Was it easy or hard to answer the questions?
- What came to mind when you were answering these questions?
- From the top of your head do you remember any statement that was difficult for you to answer? *Examples*
- How did you remember that? *Examples*

2. a Probes on specific questions of leisure-time physical activity section (A)

Q3: How often do you do VIGOROUS leisure-time physical activities for AT LEAST 10 MINUTES that cause HEAVY sweating or LARGE increases in breathing or heart rate?

- How do you understand the term ‘vigorous activity’? *Examples*
- Was it easy or hard to answer this question?
- What did ‘leisure time activity’ mean to you, in your own words?
- How did you go on answering this question?
- What came to mind when you were thinking of an answer? *Examples*

Q4: How often do you do LIGHT OR MODERATE LEISURE-TIME physical activities for AT LEAST 10 MINUTES that cause ONLY LIGHT sweating or a SLIGHT to MODERATE increase in breathing or heart rate?

- How did you come to your answer?
- What is light and what is moderate physical activity for you? *Examples*
- How do you remember this? *Examples*
- How did you decide what activities to include and exclude?

Q5: How often do you do LEISURE-TIME physical activities specifically designed to STRENGTHEN your muscles such as lifting weights or doing calisthenics?

- What does 'muscle strengthening' mean to you? *Examples*
- How would you say that in your own words?
- How did you go about answering this question? *Examples*
- Do you think that some people might not give a true answer to this question? Why?

3. General probes for the total physical activity questions (section B)

- How were these questions for you?
- Was it easy or hard to answer questions?
- What came to mind when you were answering these questions?
- From the top of your head do you remember any statement that was difficult for you to answer? *Examples*
- How did you remember that? *Examples*

3. a Probes on specific questions of total physical activity section (B)

Q6: During the last 7 days, on how many days did you do vigorous physical activities? (vigorous physical activities make you breathe much harder than normal and may include heavy lifting, digging, aerobics, or fast bicycling**)**

- How was your understanding of "vigorous activity"? *Examples*
- Was it easy or hard to answer this question?
- Did you have a particular time period in mind?
- Did you think about "a usual week" or " the last 7 days"?
- What activities did you think of when answering this question? Did you think about vigorous activity in general or did you focus on the examples?
- Do you think that some people might not give a true answer to this question? Why?

Q6a: How much time did you usually spend doing vigorous physical activities on one of those days?

- How did you go about calculating your answer? *Examples*
- How did you decide on the one day to think about?
- Was it easy or hard to answer this question?

Q8: During the last 7 days, on how many days did you walk for at least 10 minutes at a time?

- How did you decide on which walks to include? How did you decide that a walk lasted 10 minutes?
- How sure were you of your answer?
- Did you think about regular walks you do or did you also include irregular walks?

Q8a: How much time did you usually spend walking on one of those days?

- How did you go on answering this question? *Examples*
- How is your understanding of 'usually spend walking'?
- How did you decide on what to include and exclude when thinking about 'usually spend walking'?
- And how did you calculate an overall figure?

Q9: During the last 7 days, how much time did you usually spend *sitting* on a week day?

- What does the term 'sitting' in this context mean to you?
- How did you decide on what to include and exclude?
- And how did you calculate an overall figure?
- Was it easy or hard to decide what occasions to include or exclude? *Examples*
- What days were you thinking of when answering this question?

Overall probe: Interviewer please explore the kinds of physical activity the respondent usually does, or has done in the last month. Then explore if there were any activities that they did not include, or were unsure about where they should include these, and why this was.
